# Effectiveness and Nephrotoxicity of Loading Dose Colistin–Meropenem versus Loading Dose Colistin–Imipenem in the Treatment of Carbapenem-Resistant *Acinetobacter baumannii* Infection

**DOI:** 10.3390/pharmaceutics14061266

**Published:** 2022-06-14

**Authors:** Wasan Katip, Peninnah Oberdorfer, Nongyao Kasatpibal

**Affiliations:** 1 Department of Pharmaceutical Care, Faculty of Pharmacy, Chiang Mai University, Chiang Mai 50200, Thailand; 2 Epidemiology Research Group of Infectious Disease (ERGID), Chiang Mai University, Chiang Mai 50200, Thailand; pen.oberdorfer@gmail.com (P.O.); nongyaok2003@gmail.com (N.K.); 3 Division of Infectious Diseases, Department of Pediatrics, Faculty of Medicine, Chiang Mai University, Chiang Mai 50200, Thailand; 4 Division of Nursing Science, Faculty of Nursing, Chiang Mai University, Chiang Mai 50200, Thailand

**Keywords:** loading dose colistin, meropenem, imipenem, combination therapy, CRAB infections

## Abstract

Carbapenem-resistant *Acinetobacter* *baumannii* (CRAB) is becoming more widely recognized as a serious cause of nosocomial infections, and colistin has been reintroduced in recent years for the treatment of CRAB infection. Combinations of colistin and meropenem or imipenem have been found to be effective against CRAB isolates, whereas clinical investigations have not definitively demonstrated the theoretical benefits of colistin combined therapy in patients with CRAB infections. The objective of this study was to compare the primary outcome (30-day survival rate) and secondary outcomes (clinical response, microbiological response and nephrotoxicity) between patients who received loading dose (LD) colistin–meropenem and LD colistin–imipenem for the treatment of CRAB infection. A retrospective cohort analysis was performed at Chiang Mai University Hospital in patients with CRAB infection who received LD colistin–meropenem or LD colistin–imipenem between 2011 and 2017, and 379 patients fulfilled the requirements for the inclusion criteria. The results of this study showed that patients who received LD colistin–imipenem had a lower 30-day survival rate (adjusted HR = 0.57, 95% CI: 0.37–0.90; *p* = 0.015) and a lower clinical response (aHR = 0.56, 95% CI: 0.35–0.90; *p* = 0.017) compared with those who received LD colistin–meropenem. The microbiological response in patients with LD colistin–imipenem was 0.52 times (aHR) lower than that in those who received colistin–meropenem (95% CI: 0.34–0.81; *p* = 0.004); however, there was no significant difference in nephrotoxicity (aHR = 1.03, 95% CI: 0.67–1.57; *p* = 0.897) between the two combination regimens. In conclusion, when comparing the combination of LD colistin with imipenem or meropenem, the combination of LD colistin and meropenem provides a better survival rate for treating CRAB. Thus, we suggest that combinations of LD colistin and meropenem should be considered when treating CRAB infections.

## 1. Introduction

Carbapenem-resistant *Acinetobacter baumannii* (CRAB) has emerged as a significant cause of nosocomial infections in the healthcare setting, the majority of which are generally resistant to many antibiotics and only susceptible in vitro to a few antibiotics, such as colistin. Colistin inhibits the growth of most aerobic Gram-negative bacteria (GNB) in vitro [1,2] and promotes fast bacterial death in a concentration-dependent fashion [2]. In vitro studies also showed the antimicrobial efficacy of colistin against multidrug-resistant (MDR) GNB, such as *Klebsiella pneumoniae*, *Acinetobacter baumannii* and *Pseudomonas aeruginosa* [1,3]. However, physicians increasingly abandoned colistin methanesulfonate (CMS), a parenteral formulation of colistin, in the 1960s and 1970s [1] due to commonly documented dose-dependent side effects, such as nephrotoxicity. Nevertheless, the emergence of MDR bacterial infections, particularly those caused by Gram-negative organisms, is causing concern [3]. In the last decade, researchers have sought to rebuild the path that modern drugs take before entering clinical use [3]; thus, colistin has been reintroduced into clinical practice in recent years as a result of the restricted therapy choices for CRAB infections.

However, recent pharmacokinetic studies achieved plasma colistin concentrations in the range of only 1–4 mg/L after the intravenous administration of colistin in humans, suggesting that the currently recommended colistin dosage may be inadequate for eliciting an antibacterial effect when treating against pathogens with higher minimal inhibitory concentrations (MICs) [4,5]. As a result, using a loading dosage (LD) in critically ill patients is strongly recommended [4,5].

Colistin’s synergy with a variety of antibiotics, including imipenem, meropenem, rifampin, fosfomycin, sulbactam and tigecycline, has also been demonstrated in animal models [1,6,7,8]. In vitro heteroresistance has also been observed in MDR *A. baumannii* strains, despite the fact that they are expected to be colistin-sensitive [9]. Moreover, in vitro investigations have shown that colistin monotherapy can promote the regrowth of GNB [10,11]. Colistin resistance can develop during the first 24 h of treatment with colistin monotherapy, but could be inhibited and extended with combination therapy [12]. Combination therapy restricts and suppresses microbial resistance, reduces antibiotic toxicity, effectively covers a wider variety of infections and, most importantly, leads to a synergistic effect, which is convincing evidence in favour of the use of combination therapy [12,13].

Carbapenem antibiotics, such as imipenem, meropenem and doripenem, have a favourable safety profile and are still used to treat a variety of MDR GNB infections [13]. Meropenem and imipenem both cause bacterial lysis in susceptible organisms by binding to high-molecular-mass penicillin-binding proteins with high affinity [14]. CRAB has low to moderate carbapenem resistance, and the majority of isolates lack metallo-beta-lactamase production [13]. The addition of carbapenem to colistin is, therefore, expected to save its role as a combination antimicrobial agent in the battle against CRAB infections [13].

Thus, infections caused by CRAB may benefit from a combination of imipenem or meropenem and colistin. The combination of imipenem or meropenem and colistin can boost carbapenem’s efficacy against CRAB via a synergistic or additive effect, with a lower serum level of colistin, as well as diminishing colistin’s dose-dependent toxicities [1,13,15]. Combining colistin with carbapenems inhibited bacterial regrowth and reduced colistin MICs twofold [15]. Moreover, according to an in vitro investigation, combinations of colistin with ceftazidime, ceftriaxone, imipenem or meropenem had in vitro synergistic action against local CRAB strains in Vietnam [15], with colistin plus meropenem having the most synergistic potential. This was in contrast to the findings of an in vitro investigation in Thailand, where the checkerboard microdilution panel method revealed that imipenem combined with colistin had synergistic antibacterial activity against 100% of CRAB isolates [13]. Moreover, the findings of an in vitro study suggested that the maximum dose of imipenem or meropenem must be combined with colistin to obtain long-term bactericidal action against CRAB infection [13]. However, it is unknown whether meropenem or imipenem is more effective and safer when administered in combination with colistin. Furthermore, these preliminary study findings are based mainly on in vitro studies [1,13,15]. There are no real-world patient studies to support the in vitro findings. Thus, the objective of this study was to compare the effectiveness and safety of LD colistin–meropenem versus LD colistin–imipenem against CRAB infection, as well as the therapies individually.

## 2. Materials and Methods

### 2.1. Study Setting and Participants

A single-centre, retrospective cohort study on the treatment outcome of CRAB infections was conducted at Chiang Mai University Hospital (CMUH) from January 2011 to August 2017. This study was approved by the Ethics Committee on Human Research of the Faculty of Medicine, Chiang Mai University, with a waiver of informed permission for retrospective data collection on the condition that the data be anonymously preserved. All procedures were carried out in conformity with the applicable rules and regulations. The criteria for identifying and classifying infections were developed by the US Centers for Disease Control and Prevention (CDC) [16] and were based on the opinions of infectious disease (ID) physicians. Patients who were 18 years old, received colistin for more than 2 days to treat a documented CRAB infection and received only one round of colistin treatment were included in the study. Only patients who had not received any other treatment with possible anti-*A. baumannii* action were included. Patients having colonisers or contaminants in their CRAB cultures, as well as those with incomplete records, were excluded. The patients were split into two groups: those who received LD colistin–meropenem and those who received LD colistin–imipenem. Treatment with meropenem or imipenem in combination with colistin for more than 48 h was the inclusion criterion for patients in the combined therapy group. Colistin was administered intravenously (i.v.) as a LD of 300 mg (9 million units of CMS), followed by 150 mg of colistin base activity (CBA) every 12 h (corrected according to renal function). Meropenem was administered in a 1000 mg i.v. dose over 0.5 h every 8 h, while imipenem was administered in a 500 mg i.v. dose over 0.5 h every 6 h.

### 2.2. Data Collection

Patient information was obtained through the use of computerized medical records and a review of patient charts. Age, sex, diagnosis, underlying diseases, serum creatinine, creatinine clearance, duration of LD colistin–meropenem or –imipenem concomitant therapy, microbial culture, source of infection (documented by treating physicians), LD colistin–meropenem or –imipenem daily dose, length of hospital stay, colistin–meropenem or –imipenem concomitant duration, timing of antibiotic therapy, mortality, clinical outcome, APACHE II score obtained on the day of admission to the hospital and nephrotoxicity were all collected.

### 2.3. Evaluation of the Outcome

The primary outcome in this study was the 30-day survival rate: the survival rate within 30 days of CRAB infection was characterized as the 30-day survival. Secondary outcomes included the clinical response at the end of treatment and the microbiological response. The clinical response was measured by the resolution or partial resolution of fever, leucocytosis and local signs and symptoms of CRAB infection after the completion of antibiotic therapy. Clinical failure was defined as failing to achieve all clinical response criteria. Microbiological response was defined as two consecutive negative CRAB cultures from the site of infection after the initial positive culture, whereas microbiological failure was defined as the persistence of the same causal organism in subsequent specimen cultures. The safety outcome was evaluated at the end of antibiotic treatment. If patients experienced any degrees of renal failure based on RIFLE criteria, nephrotoxicity was counted.

### 2.4. Antimicrobial Susceptibility Testing

All causal bacteria were identified using standard microbiological techniques. The disk diffusion method and an automated broth microdilution method (VITEK 2 system, bioMérieux, Marcy-l’Étoile, France) were used to evaluate susceptibility. The Clinical and Laboratory Standards Institute (CLSI) procedure [17] was used to assess antimicrobial susceptibility. The VITEK 2 system was used to test *A. baumannii* antibiotic susceptibility to colistin, with resistance defined as a colistin minimum inhibitory concentration (MIC) breakpoint >2 mg/L. The VITEK 2 system is a completely automated system that uses fluorogenic technology to identify organisms to measure susceptibility [18]. *A. baumannii* was classified as CRAB if it was resistant to carbapenems but susceptible to colistin.

### 2.5. Statistical Analysis

Stata software version 14 was used to conduct the statistical analysis (Stata-Corp, College Station, TX, USA). Continuous data were given as means and standard deviations, while categorical variables were described as frequencies and percentages. Fisher’s exact test was employed for categorical data, and an independent *t*-test was utilized for continuous variables to compare two groups. A statistically significant result was defined as a two-tailed test with a *p*-value of less than 0.05. Fisher’s exact test was used to assess differences in the crude primary outcome rates (30-day survival rate) and secondary outcomes (clinical response, microbiological response and nephrotoxicity) between the two groups.

In order to decrease potential biases, inverse-probability-weighted (IPW) propensity score modification was conducted due to imbalances in the baseline characteristics of the treatment groups. The inverse probability of treatment weighting is the use of probability weights to reduce the imbalance in potential confounding factors between treated and control patients. Using multivariable logistic regression, we obtained the propensity score. The variables used to calculate the propensity score were likely to influence the outcomes (Charlson score) and baseline covariates with an inclusion criterion of *p*-value < 0.1 [19] (hypertension, cardiovascular disease, diabetes mellitus, chronic obstructive pulmonary disease, malignancy, chronic liver disease and septic shock). However, some covariates (such as chronic liver disease and aminoglycosides) had a small number of cases and were, therefore, not included to calculate the propensity score.

The weights were then used to examine the outcomes for the two treatment groups using cause-specific Cox proportional hazards regression models to account for the data’s time to event nature.

Variables related to both the primary (30-day survival) and secondary (i.e., clinical response, microbiological response and nephrotoxicity) outcomes were investigated using a univariate Cox regression analysis. Cox proportional hazards regression analysis was used to analyse the multiple variable analysis to estimate the adjusted hazard ratios (HR) and 95% confidence intervals (CI) of relevant components (inverse probability weighting using the propensity score for baseline covariate adjustment). For all analyses, two-sided α = 0.05 was considered statistically significant.

## 3. Results

During the study period, 379 adult patients, who were admitted to the CMUH and fulfilled the inclusion criteria, were reviewed and recruited: 311 patients (82.06%) were treated with LD colistin–meropenem and 68 patients (17.94%) with LD colistin–imipenem. Of the total number of patients, 225 cases (59.37%) were female, and the mean ± SD age was 65.47 ± 17.21 years. Hypertension, cardiovascular disease, diabetes mellitus and chronic renal disease were the most frequent underlying diseases. Table 1 shows the study patients’ characteristics, as well as comparisons between LD colistin–meropenem and LD colistin–imipenem.

In a rough comparison of the 30-day survival rates, 53.70% of patients received LD colistin–meropenem and 48.53% received LD colistin–imipenem (*p* = 0.503). A clinical response was reported in 54.66% of patients in the LD colistin–meropenem group and 44.12% of patients in the LD colistin–imipenem group (*p* = 0.140), and the rate of microbiological response in the two groups was 62.38% and 54.41%, respectively (*p* = 0.272). Furthermore, according to the RIFLE criteria, the rates of nephrotoxicity in the LD colistin–meropenem and LD colistin–imipenem groups were 44.37% and 60.29%, respectively (*p* = 0.022). Table 2 shows the results of the crude analysis.

**Figure 1:**
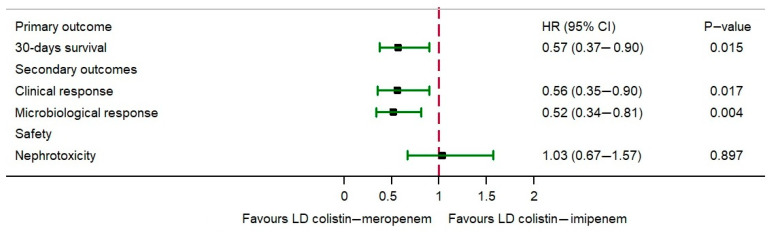
Forest plot of hazard ratios and 95% confidence intervals of outcomes for patients with CRAB infection receiving LD colistin–meropenem and LD colistin–imipenem therapy.

Univariate Cox regression analysis showed that the LD colistin–imipenem group was not associated with the 30-day survival rate compared with the LD colistin–meropenem group (HR: 0.78, 95% CI: 0.54–1.14; *p* = 0.211). Furthermore, the clinical response (HR: 0.70, 95% CI: 0.47–1.03; *p* = 0.075), microbiological response (HR: 0.75, 95% CI: 0.53–1.07; *p* = 0.123) and nephrotoxicity were not different between patients who received LD colistin–meropenem and those who received LD colistin–imipenem (HR: 1.18, 95% CI: 0.84–1.68; *p* = 0.343). The multivariate Cox proportional hazards model (IPW using the propensity score) adjusted for variables found a significant association with the 30-day survival rate (primary outcome), with patients who received LD colistin–imipenem having a lower 30-day survival rate than those who received LD colistin–meropenem (HR = 0.57, 95% CI:0.37–0.90; *p* = 0.031). Moreover, the secondary outcomes of the IPW propensity score analysis using the Cox regression model showed that LD colistin–imipenem was associated with a significant decrease in clinical response (aHR: 0.63, 95% CI: 0.37–1.08; *p* = 0.096) and microbiological response (aHR: 0.52, 95% CI: 0.34–0.81; *p* = 0.004) compared with LD colistin–meropenem. However, the nephrotoxicity rate of LD colistin–imipenem showed no difference (aHR: 1.03, 95% CI: 0.67–1.57; *p* = 0.897) compared with LD colistin–meropenem. All outcomes for patients with CRAB infections receiving LD colistin–meropenem and LD colistin–imipenem therapy are shown in Figure 1. In a subgroup analysis of patients with and without previous chronic kidney disease (CKD), the colistin–imipenem group showed no difference in the nephrotoxicity rate compared with those receiving LD colistin–meropenem (Table 3).

## 4. Discussion

Based on the results of this retrospective study, the combination of LD colistin–meropenem resulted in a higher 30-day survival rate when compared with LD colistin–imipenem. Furthermore, LD colistin–meropenem was associated with significantly higher clinical responses and microbiological responses when compared with LD colistin–imipenem. However, nephrotoxicity was not significantly associated with LD colistin–meropenem when compared with LD colistin–imipenem. The addition of meropenem to LD colistin is expected to become the antibacterial of choice in combat against CRAB infections.

Antimicrobial combinations have the potential to improve outcomes by expanding the spectrum of antimicrobial activity, reducing the risk of antimicrobial resistance and producing a stronger antimicrobial effect via synergy [12]. Because of probable variations in the pharmacokinetic effects of these medications in the host, as well as varying bacterial concentrations and drug concentrations at different infection sites, clinical efficacy cannot be assumed. Clinical research is needed to clarify the therapeutic potential of this combination, and an appropriate combination of these medications should be thoroughly evaluated in clinical studies of CRAB infections [12].

Colistin-based combinations have been recommended to prevent treatment failure caused by the formation of polymyxin-resistant *A. baumannii* during therapy, and suboptimal pharmacokinetics and pharmacodynamics [20,21]. The addition of carbapenem may help to prevent or diminish the emergence of resistant mutants during antibiotic treatment, as regrowth during 24 h of incubation was less with this combination [13]. An in vitro growth dynamics investigation also showed significant decreases in *P. aeruginosa* regrowth with a colistin–carbapenem combination, as well as a delay in the formation of colistin-resistant subpopulations [22,23]. The proposed mechanism to explain the synergy between colistin and carbapenem indicates that colistin increases the permeability of the bacterial outer membrane, allowing more carbapenem to enter the bacteria [24]. Furthermore, larger concentrations of carbapenem can decrease the resistance mechanisms of the bacteria, making carbapenem more effective against drug-resistant bacteria [24].

Consistent with prior laboratory findings, our investigation found a difference in the 30-day survival, clinical response and microbiological response between patients with CRAB infection who were treated with LD colistin–meropenem and those treated with LD colistin–imipenem. The thirty-day survival in the LD colistin–imipenem group was 0.57 times (aHR) lower compared with that of the LD colistin–meropenem group (95% CI: 0.37–0.90; *p* = 0.015). The clinical response in the LD colistin–imipenem group was 0.56 times (aHR) lower compared with that of LD colistin–meropenem (95% CI: 0.35–0.90; *p* = 0.017) and the microbiological response in patients with LD colistin–imipenem was 0.52 times (aHR) lower than that in those receiving colistin–meropenem (95% CI: 0.34–0.81; *p* = 0.004). Several reasons might explain these phenomena: (1) The relatively low dose of imipenem (500 mg i.v. over 0.5 h every 6 h) in this study may have led to insufficient concentration levels of imipenem at the site of infection, resulting in a poor synergistic effect [25]. (2) The in vitro study by Song et al. found that colistin plus imipenem did not significantly reduce the bacterial burden in the lungs after infection [26], possibly because the colistin–imipenem combination did not work as well as expected. (3) The MICs of meropenem against CRAB were not extremely high in our study, being less than 32 mg/L against a total of 311 CRAB isolates in the colistin–meropenem group in our investigation; therefore, a synergistic effect was seen in our study. An in vitro investigation by Fan et al. [27] found that colistin–meropenem combination therapy had synergistic effects and was superior to colistin plus tigecycline, fosfomycin or sulbactam in strains with low meropenem MICs (≤32 mg/L), thus supporting our findings. However, no synergistic effects were identified in this combination therapy with strains with higher MICs (≥64 mg/L) [27]. (4) According to one probable mechanism, meropenem has a greater affinity for penicillin-binding proteins in GNB than imipenem [12]. (5) It is possible that the enhanced synergy with meropenem over imipenem is due to the fact that most carbapenemases target imipenem with greater affinity compared with meropenem [28]. Thus, synergism was observed more frequently in LD colistin–meropenem than in LD colistin–imipenem, which could explain why meropenem performed better in our investigation.

Our findings are supported by a systematic review and meta-analysis of the in vitro synergy of polymyxins and carbapenems [29]. This study used all papers involving in vitro interactions between antibiotic combinations containing any carbapenem and colistin or polymyxin B and any GNB. In a mixed-effect meta-analysis of rates, synergy rates, which were defined as a fractional inhibitory concentration index of 0.5 or a >2-log reduction in CFU, were pooled individually for time–kill, checkerboard and E-test techniques, and the rates were provided with their 95% CI. There were 39 published publications and 15 conference proceedings in total, with 246 distinct tests reported on 1054 bacterial isolates. The time–kill analysis was carried out on 186 isolates of *A. baumannii* and found that meropenem was more synergistic with colistin than imipenem for *A. baumannii* (*p* = 0.008 for subgroup comparison) [29]. Moreover, our findings agree with a previous systematic review and meta-analysis of the in vitro synergy of antibiotic combinations against *A. baumannii* assessed by pharmacokinetic/pharmacodynamic and time–kill studies, which indicated significant benefits for *A. baumannii* infections when meropenem was combined with colistin, rather than imipenem [30].

Our findings are also consistent with those from another in vitro study by Soudeiha et al., which evaluated the efficacy of colistin–carbapenem combinations against *Acinetobacter* spp., with the goal of decreasing the requirement for high antibiotic doses in therapy. This analysis was performed on 100 non-duplicate *Acinetobacter* isolates taken from various patients at Beirut’s Saint George Hospital–University Medical Center. All approaches revealed an additive impact for the colistin–carbapenem combination, and Soudeiha et al. found that the combination of colistin–meropenem had better outcomes and additive impact than colistin–imipenem (*p* < 0.05) [15]. 

Moreover, the in vitro activity of colistin in combination with fosfomycin or imipenem was evaluated against eight CRAB strains by Santimaleeworagun et al. Between January and December 2008, eight CRAB clinical isolates were recovered from hospitalized patients at Songklanagarind Hospital in southern Thailand. Colistin was totally susceptible to all isolates producing OXA-23 carbapenemases, although other antimicrobial drugs were only occasionally susceptible, and no synergy was found between colistin and imipenem [31]. 

One of the most typically seen side effects of colistin intravenous injection is nephrotoxicity, which makes it the dose-limiting factor. However, when comparing LD colistin–meropenem and LD colistin–imipenem combination therapy, we found no significant difference in nephrotoxicity (aHR = 1.03, 95% CI: 0.67–1.57; *p* = 0.897). Moreover, the colistin–imipenem group exhibited no difference in the nephrotoxicity rate when compared with the LD colistin–meropenem group in a subgroup study of patients with and without preexisting CKD. This result might be explained by our study not involving an increase in the colistin dose, so it was impossible to demonstrate the dose-limiting factor mentioned above. Additionally, GFR and SCr were used to calculate the propensity score when multivariable logistic regression was performed. Therefore, there should be no significant difference in nephrotoxicity. 

Very few head-to-head comparison clinical studies of a combination of LD colistin with meropenem or imipenem have been conducted. This large retrospective study provides evidence regarding the effectiveness of colistin combinations in the treatment of CRAB infection. Thus, before more randomised studies are carried out, our findings suggest that the colistin–meropenem combination may be used for the treatment of CRAB infection.

There are several limitations to the present study. Firstly, we identified significant variations in the baseline characteristics between the therapy groups, despite the fact that this difference was also detected in other retrospective investigations and was difficult to match in both groups, potentially leading to confounding. To account for known baseline features, an IPW propensity score method was applied. Furthermore, in order to address the most significant confounders, we performed a multivariable Cox regression analysis to ascertain that statistically significant confounders with clinical validity remained in our final multivariable model. However, it is unlikely that the fundamental differences can be completely compensated. As a result, the findings should be regarded with caution because the baseline state differed, which could have a significant impact on the survival rate. Secondly, because the trial was conducted at a single location, the clinical features, CRAB strains and the prevalence of genetic resistance mechanisms may differ depending on the local epidemiology, thereby affecting the outcome of the combination therapy. Thirdly, synergy is not general and does not apply to all *A. baumannii* strains. For strains with extremely high MICs, clinically significant synergy may be less likely. For isolates with high carbapenem MICs (doripenem >64 mg/L, meropenem ≥64 mg/L), clinically meaningful synergy between colistin and carbapenems appears to be less likely [24]. Finally, our study had a short data collection time. If the collection time in future studies can be extended, the number of patients in each group can be increased, resulting in more robust results.

## 5. Conclusions

In this study, we found that LD colistin–imipenem combination therapy significantly decreased the 30-day survival rate during CRAB infections when compared with patients receiving LD colistin–meropenem treatment, while also reducing the clinical response and microbiological response compared with LD colistin–meropenem. However, there was no significant difference in the nephrotoxicity between the two combination regimens. Our findings suggest that combining LD colistin with meropenem could be a promising therapeutic strategy for treating CRAB infections.

## Figures and Tables

**Table 1:** Demographic and clinical characteristics of patients who received LD colistin–meropenem compared with LD colistin–imipenem therapy.

Characteristic	Colistin–Meropenem (*n* = 311)	Colistin–Imipenem (*n* = 68)	*p*-Value
Sex, *n* (%)			
Male	129 (41.48)	25 (36.76)	0.499
Female	182 (58.52)	43 (63.24)	
Age, years, mean ± SD	65.98 ± 17.52	63.14 ± 15.60	0.218
Duration of treatment, days, mean ± SD	9.20 ± 6.17	10.04 ± 5.73	0.307
Comorbidities *, *n* (%)			
Hypertension	149 (47.91)	24 (35.29)	0.061
Cardiovascular disease	113 (36.45)	13 (19.12)	0.007
Diabetes mellitus	76 (24.44)	9 (13.24)	0.053
Chronic kidney disease	72 (23.23)	13 (19.12)	0.524
Chronic obstructive pulmonary disease	57 (18.33)	6 (8.82)	0.071
Malignancy	72 (23.15)	25 (36.76)	0.031
Chronic liver disease	21 (6.77)	1 (1.47)	0.147
Septic shock	229 (73.63)	41 (60.29)	0.038
Mechanical ventilation	216 (83.92)	56 (82.35)	0.720
Charlson score, median, mean ± SD	2.69 ± 2.26	2.29 ± 1.72	0.167
APACHE II score ^a^, mean ± SD	7.94 ± 4.29	7.91 ± 3.83	0.948
ICU admission	227 (72.99)	44 (64.71)	0.183
Baseline SCr, mg/dL, median (IQR)	0.90 (0.60–1.50)	0.80 (0.50–1.50)	0.175
Baseline GFR, mL/min, median (IQR)	54.59 (18.57–95.74)	69.27 (33.45–103.55)	0.101
Total colistin dose, g, median (IQR)	1.60 (0.90–2.85)	1.85 (1.05–3.12)	0.175
Type of nephrotoxic medications ^#^, *n* (%)			
Aminoglycosides	2 (0.64)	3 (4.41)	0.042
Diuretics	245 (78.78)	57 (83.82)	0.408
Amphotericin B	28 (9.00)	11 (16.18)	0.120
Vasopressors	226 (72.67)	43 (63.24)	0.140
Vancomycin	199 (63.99)	37 (54.41)	0.167
Site of CRAB infection			
Pneumonia	260 (83.60)	53 (77.94)	0.290
Bacteraemia	15 (4.82)	4 (5.88)	0.758
UTI	34 (10.93)	9 (13.24)	0.535
Other *	17 (5.47)	5 (7.35)	0.567
MIC colistin µg/mL, median (min–max)	0.50 (0.50–1.00)	0.50 (0.50–1.00)	1.000

SCr, serum creatinine; GFR, glomerular filtration rate; SD, standard deviation; UTI, urinary tract infection; ^a^, at the time of admission; *, intercostal drainage and surgical site infection; IQR, interquartile range; ^#^, each patient could have had more than one drug.

**Table 2:** Primary and secondary outcomes for patients who received LD colistin–meropenem compared with LD colistin–imipenem therapy.

Outcomes	Colistin–Meropenem (*n* = 311)	Colistin–Imipenem (*n* = 68)	*p*-Value
**Primary outcome**			
30-day survival rate	167 (53.70)	33 (48.53)	0.503
**Secondary outcomes**			
Clinical response	170 (54.66)	30 (44.12)	0.140
Microbiological response	194 (62.38)	37 (54.41)	0.272
**Safety**			
Nephrotoxicity	138 (44.37)	41 (60.29)	0.022

**Table 3:** Cox regression analysis of safety for CRAB infection between LD colistin–meropenem compared to LD colistin–imipenem therapy.

Outcome and Variable *	Colistin–Meropenem (*n* = 311)	Colistin–Imipenem (*n* = 68)	Crude HR (95% CI)	*p*-Value	Adjusted HR ** (95% CI)	*p*-Value
**Safety**						
**Nephrotoxicity**	138 (44.37)	41 (60.29)	1.18 (0.84–1.68)	0.343	1.03 (0.67–1.57)	0.897
- Previous CKD	19 (26.39)	3 (26.39)	0.87 (0.26–2.94)	0.821	0.02 (0.01–3.42)	0.268
- Non-CKD	119 (49.79)	38 (69.09)	1.19 (0.83–1.72)	0.347	1.38 (0.88–2.15)	0.159

CKD, chronic kidney disease; CI, confidence interval; ** Inverse probability weighting (IPW) using the propensity score for baseline covariate adjustment; HR, hazard ratio; * LD colistin–meropenem (reference).

## Data Availability

The datasets used and analysed during the current study are available from the corresponding author on reasonable request.
